# Bi-directional metabolic reprogramming between cancer cells and T cells reshapes the anti-tumor immune response

**DOI:** 10.1371/journal.pbio.3003284

**Published:** 2025-07-14

**Authors:** Yajing Qiu, Yihan Xu, Xinyuan Ding, Congcong Zhao, Hongcheng Cheng, Guideng Li

**Affiliations:** 1 National Key Laboratory of Immunity and Inflammation, Suzhou Institute of Systems Medicine, Chinese Academy of Medical Sciences & Peking Union Medical College, Suzhou, Jiangsu, China; 2 Key Laboratory of Synthetic Biology Regulatory Elements, Suzhou Institute of Systems Medicine, Chinese Academy of Medical Sciences & Peking Union Medical College, Suzhou, Jiangsu, China; 3 Department of Pharmacy, Medical Science University and Suzhou Technology China Innovation Center, The Affiliated Suzhou Hospital of Nanjing Medical University, Suzhou Municipal Hospital, Gusu School of Nanjing Medical University, Suzhou, China; Princeton University, UNITED STATES OF AMERICA

## Abstract

Cancer cells and T cells engage in dynamic crosstalk within the tumor microenvironment (TME), shaping tumor progression and anti-tumor immunity. While cancer cells reprogram metabolism to support growth and immune evasion, T cells must adapt their metabolic states to maintain effector functions. Tumor-driven metabolic perturbations, such as nutrient depletion and accumulation of immunosuppressive metabolites, profoundly impair T cell function and fate. Conversely, metabolically reprogrammed T cells can modulate the TME and influence tumor growth. This reciprocal metabolic crosstalk represents both metabolic competition and intercellular communication, offering promising therapeutic targets.

## Abstract 2

Cancer cells and T cells engage in dynamic metabolic competition and adaptation within the tumor microenvironment. This Essay discusses bi-directional metabolic crosstalk between tumors and T cells, highlighting metabolic targeting as a promising strategy to reprogram anti-tumor immune responses.

## Introduction

Cancer is not only a genetic disease but also a profoundly metabolic one [1]. Within the tumor microenvironment (TME), a dynamic ecosystem composed of cancer cells, immune cells, fibroblasts and vasculature, metabolic reprogramming fuels tumor progression while actively shaping immune responses [2,3]. To meet their high demands for energy and biosynthesis, tumor cells up-regulate glycolysis, glutaminolysis and lipid metabolism. These adaptations support proliferation and survival but simultaneously create a hostile microenvironment defined by nutrient depletion, hypoxia and acidosis [3–9]. Such metabolic remodeling enables immune evasion while directly suppressing the function of cytotoxic T cells [10–13]. Through nutrient competition, tumor cells deprive T cells of glucose, glutamine and amino acids, impairing their metabolic fitness, cytokine production and effector activity [7–9,14–16]. Meanwhile, acidification and hypoxia, driven by hypoxia-inducible factor 1α (HIF-1α), favor the accumulation of regulatory T cells (Tregs) and up-regulate checkpoint molecules, further dampening immune surveillance [17–19]. The acidification of the TME in particular is largely due to lactate accumulation, which disrupts T cell proliferation and viability [20,21]. Importantly, T cells themselves are metabolically responsive. In the TME, they undergo substantial metabolic rewiring, which governs their activation, lineage fate and persistence [22–24]. Tregs, in particular, exhibit a metabolic profile well-suited to this hostile environment, relying on oxidative phosphorylation and fatty acid oxidation (FAO) to maintain their suppressive function under hypoxic and nutrient-depleted conditions [23,25–27].

This bidirectional metabolic crosstalk has emerged as a key driver of tumor immune escape and therapy resistance. In response, new therapeutic strategies are being developed that target tumor metabolism while restoring T cell function, such as inhibitors of lactate production and glutaminase activity, metabolic adjuvants (e.g., taurine, acetate) and combination regimens that integrate metabolic reprogramming with immune checkpoint blockade [28–32]. Advances in spatial metabolomics and single-cell multiomics offer powerful tools to dissect this complexity and guide context-specific interventions [33,34].

In this Essay, we discuss how metabolic rewiring in both tumor cells and T cells reshapes the immune landscape and contributes to malignant progression. We highlight emerging strategies that aim to break the metabolic stalemate, opening avenues for precision cancer immunotherapy that targets both metabolism and immunity.

## Metabolic reprogramming in cancer cells

Metabolic reprogramming is recognized as a hallmark of cancer, in which tumor cells reconfigure their metabolic networks to support uncontrolled proliferation, invasion, metastasis and resistance to various forms of regulated cell death (including apoptosis, ferroptosis, pyroptosis and others) [1,35–40]. In this section, we focus on carbohydrate, amino acid and lipid metabolism in cancer cells to explore how these metabolic pathways contribute to tumor progression and therapy resistance.

### Altered carbohydrate metabolism

**Fig 1:**
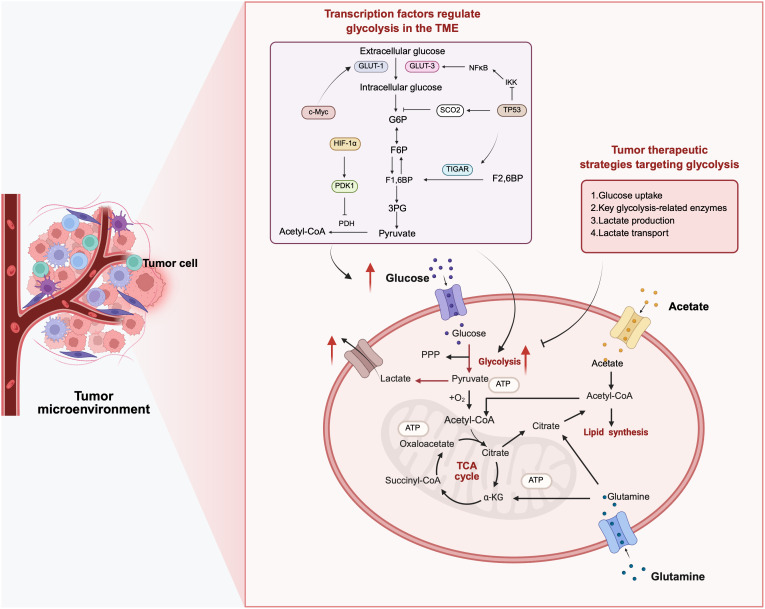
Metabolic reprogramming in cancer cells. Glucose metabolism involves both glycolysis and the mitochondrial tricarboxylic acid (TCA) cycle. Cancer cells preferentially adopt aerobic glycolysis, enabling rapid energy production and biosynthesis while contributing to immune evasion. To improve the effectiveness of immunotherapy, strategies to reprogram the tumor microenvironment (TME) are under investigation. Four key intervention points within the glycolytic pathway have been identified: targeting glucose uptake, inhibiting critical glycolytic enzymes, blocking lactate production and blocking lactate export. 3PG, 3-phosphoglyceric acid; ATP, adenosine triphosphate; CoA, coenzyme-A; F1,6 BP, fructose 1,6-bisphosphate; F6P, fructose 6-phosphate; F2,6 BP, fructose 2,6-bisphosphate; G6P, glucose 6-phosphate; α-KG, α-ketoglutarate; PDH, pyruvate dehydrogenase; PPP, pentose phosphate pathway. Figure created with BioRender, https://www.biorender.com.

Glucose is the primary carbohydrate fuel for both normal and malignant cells. In healthy cells, glucose metabolism is mainly metabolized through mitochondrial oxidative phosphorylation (OXPHOS), an energy-efficient process for adenosine triphosphate (ATP) generation [12]. By contrast, many cancer cells elevate glycolysis even under aerobic conditions, known as the “Warburg effect”. This metabolic shift enables rapid production of ATP and biosynthetic intermediates, while also contributing to the acidification of TME, thereby promoting angiogenesis and facilitating immune evasion (Fig 1) [41–44]. Importantly, this reprogramming is tightly linked to multiple signaling pathways that affect tumor growth, invasion and even resistance to chemotherapy [45]. For instance, in colorectal cancer (CRC), B7-H3 enhances glucose uptake and lactate production by upregulating hexokinase 2 (HK2), a critical mediator of B7-H3-induced chemoresistance in CRC cells [46]. Additionally, lactate has been implicated in promoting angiogenesis by inducing the phosphorylation and subsequent degradation of the transcription factor IκBα, thereby activating an autocrine NFκB–IL-8/CXCL8 signaling pathway [47]. While the exact molecular mechanisms linking aerobic glycolysis to chemoresistance in CRC remain to be fully elucidated, one prevailing hypothesis suggests that the acidic microenvironment and glycolysis-derived metabolic intermediates may influence drug uptake or trigger pro-survival signaling pathways, ultimately reducing the effectiveness of chemotherapeutic agents.

Not all tumors up-regulate glycolysis via the same mechanisms. In esophageal squamous cell carcinoma, enhanced aerobic glycolysis has been attributed to the E3 ubiquitin ligase TRIM33, which mediates p53 degradation through K48-linked ubiquitination. The loss of regulatory control by p53 leads to increased expression of glycolytic enzymes, including glucose transporter 1 (GLUT-1), HK2, pyruvate kinase M2 (PKM2) and lactate dehydrogenase A (LDHA), thereby fueling tumor growth [47]. In parallel, HIF-1α, which is often stabilized under low-oxygen conditions or activated by oncogenic signaling pathways (e.g., PI3K–AKT, RAS), directly upregulates glycolytic enzymes (e.g., HK2, LDHA, pyruvate dehydrogenase kinase 1) and suppresses mitochondrial pyruvate oxidation [48,49]. This dual role reinforces the glycolytic phenotype and adapts the tumor to fluctuations of oxygen in its environment. Moreover, other signaling axes, such as the IRF2–CENP-N/AKT pathway identified in nasopharyngeal carcinoma, further enhance glycolysis and contribute to the inhibition of apoptotic processes [50].

Beyond glycolysis, alternative carbohydrate pathways also contribute to tumorigenesis. For example, in triple-negative breast cancer (TNBC), impaired mannose metabolism, resulting from reduced expression of guanosine diphosphate (GDP)-mannose pyrophosphorylase A, leads to an accumulation of GDP-mannose. This buildup has been implicated in promoting the degradation of the tumor suppressor BRCA2, which in turn compromises homologous recombination and may increase genomic instability [51]. Such findings highlight that metabolic dysregulation can affect not only energy production but also critical processes such as DNA repair. Furthermore, the interplay between glycolysis and gluconeogenesis adds an additional layer of metabolic complexity. In soft tissue sarcomas, the gluconeogenic enzyme fructose-1,6-bisphosphatase 2 (FBP2) impedes tumor growth through two distinct mechanisms [52]. First, by attenuating the Warburg effect, FBP2 reduces the dependence of tumor cells on glucose metabolism [53]. Second, through a noncanonical interaction with the oncogenic transcription factor c-Myc, FBP2 suppresses c-Myc-dependent expression of mitochondrial transcription factor A, thereby diminishing mitochondrial biogenesis and respiratory activity [52]. This dual function underscores the potential of targeting metabolic flexibility as a therapeutic strategy.

### Reprogramming of amino acid and lipid metabolism

Cancer cells not only rely on enhanced glycolysis but also rewire amino acid and lipid metabolism to meet their high energetic and biosynthetic demands, which in turn supports survival, proliferation and metastatic progression (Fig 1) [12]. For example, in pancreatic ductal adenocarcinoma, succinylation at lysine 311 of kidney-type glutaminase boosts its enzymatic activity, enhancing glutaminolysis and increasing the production of nicotinamide adenine dinucleotide phosphate and glutathione, which in turn supports tumor growth [32]. Moreover, oncogenic factors such as c-Myc can further stimulate the serine synthesis pathway by upregulating enzymes including phosphoglycerate dehydrogenase, phosphoserine aminotransferase 1 and phosphoserine phosphatase. These enzymes not only contribute to de novo serine biosynthesis, which is essential for nucleotide and protein synthesis, but also bolster redox homeostasis by supporting the synthesis of glutathione and other antioxidants [54]. This highlights the importance of amino acid metabolism reprogramming in both supporting rapid cell proliferation and protecting against oxidative damage.

In parallel, cancer cells considerably alter lipid metabolism to facilitate tumor progression, particularly under nutrient-limited conditions. The transcription factor SREBF1 is a key regulator of this process, promoting the expression of lipogenic genes, including *NPC2*. This activation leads to the mobilization of cholesterol and fatty acids from stored lipid droplets, which are then utilized for membrane biosynthesis and energy production, both of which are crucial for supporting rapid tumor cell division [43]. In aggressive cancers such as hepatocellular carcinoma and metastatic breast cancer, acyl-coenzyme A synthetase long-chain family member 4 (ACSL4) has a dual role. ACSL4 enhances FAO to meet high energy demands while also influencing epithelial–mesenchymal transition (EMT) through interactions with transcriptional regulators such as ZEB2, mediated by the c-Myc–SREBP1 pathway [55–58]. This interplay between lipid metabolism and signaling pathways contributes to increased invasiveness and metastatic potential, underscoring the complex impact of lipid metabolic reprogramming on cancer progression.

The convergence of these altered metabolic pathways not only addresses the increased need for energy and biosynthetic precursors but also creates unique metabolic vulnerabilities in cancer cells. These vulnerabilities present potential therapeutic targets. For example, inhibitors of glutaminase or fatty acid synthase, along with lactate transporter blockers, are currently being explored as strategies to disrupt the metabolic balance in tumors [59–64]. Such targeted interventions could sensitize cancer cells to conventional therapies and enhance the effectiveness of immunotherapies by reprogramming the TME. Future research will be essential to better understand the specific metabolic profiles across different cancer subtypes, a key step in advancing precision medicine approaches that exploit these metabolic dependencies.

## Metabolic characteristics of T cells

**Fig 2:**
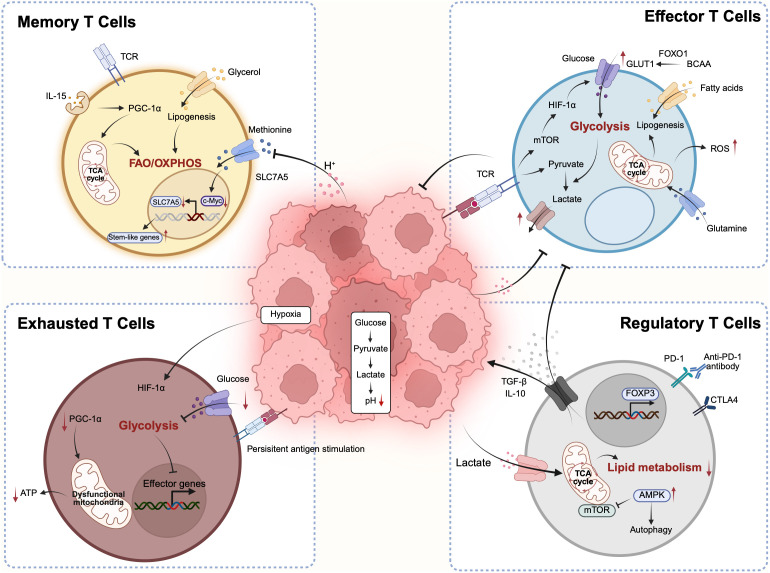
Metabolic characteristics during T cell differentiation. Upon activation, naïve T cells undergo metabolic reprogramming and differentiate into effector T cells, shifting toward aerobic glycolysis to support rapid proliferation and effector functions. This transition is accompanied by epigenetic modifications at key gene loci. Following antigen clearance, a subset of T cells differentiates into memory T cells, which revert to utilizing fatty acid oxidation (FAO) and oxidative phosphorylation (OXPHOS) to support long-term survival and functional readiness. By contrast, during chronic antigen stimulation, effector T cells are driven toward an exhausted state, characterized by metabolic reprogramming, mitochondrial dysfunction and impaired effector function. Regulatory T cells (Tregs), an immunosuppressive population that supports tumor progression, rely on OXPHOS and FAO for survival and exert their function through the secretion of inhibitory cytokines such as TGF-β and IL-10. ATP, adenosine triphosphate; BCAA, branched-chain amino acid; CTLA4, cytotoxic T-lymphocyte associated protein 4; FOXO1, forkhead box O1; FOXP3, forkhead box P3; GLUT-1, glucose transporter 1; HIF-1α, hypoxia-inducing factor 1α; mTOR, mammalian target of rapamycin; PD-1, programmed cell death protein 1; PGC-1α, peroxisome proliferator-activated receptor-γ coactivator 1-α; ROS, reactive oxygen species; TCA, tricarboxylic acid cycle; TCR, T cell receptor. Figure created with BioRender, https://www.biorender.com.

Similar to cancer cells, T cells also undergo metabolic reprogramming, and their metabolic state is closely linked to their functional status. For instance, naïve T cells maintain longevity primarily through oxidative metabolism, whereas activated effector T cells rapidly shift to glycolysis to support their proliferation and cytokine production. These distinct metabolic programs play a pivotal role in determining T cell fate, persistence and their capacity to mount effective anti-tumor immune responses (Fig 2) [65–67].

### Carbohydrate metabolism

Glucose metabolism is central to T cell activation, differentiation and function. In quiescent naïve and memory T cells, energy demands are relatively low and are primarily met through mitochondrial OXPHOS. Upon antigen recognition however, T cells undergo a metabolic switch to aerobic glycolysis to support rapid proliferation and acquisition of effector function [65,66]. This reprogramming is initiated by T cell receptor (TCR) stimulation, which upregulates the GLUT-1, thereby enhancing glucose uptake (Fig 2) [68]. The imported glucose is converted to pyruvate, which either enter the mitochondria to fuel the tricarboxylic acid (TCA) cycle or is reduced to lactate in the cytosol. TCR signaling also induces the expression of pyruvate dehydrogenase kinase 1, which inhibits the entry of pyruvate into mitochondria, promoting lactate production and enhancing glycolytic flux to sustain T cell activation and function [69,70]. Co-stimulatory signals, particularly through CD28, further amplify GLUT-1 expression and glucose uptake, reinforcing the glycolytic program [71]. The transcription factor c-Myc, which is rapidly induced upon TCR engagement, serves as a master regulator of this process by promoting the expression of glycolytic genes, including glucose transporters and key enzymes such as HK2 and LDHA. Moreover, glycolytic intermediates fuel anabolic pathways such as the pentose phosphate pathway (PPP) and nucleotide biosynthesis, which are essential for biomass accumulation and clonal expansion of proliferating T cells [65,72,73].

By contrast, memory T cells down-regulate mTOR signaling and glycolysis, relying instead on mitochondrial metabolism and FAO for long-term survival. Inhibiting glycolysis during activation, for instance with 2-deoxyglucose, promotes memory T cell formation [74,75]. Glucose restriction also promotes AMP-activated protein kinase (AMPK)-mediated activation of the SENP1–SIRT3 axis, thereby enhancing OXPHOS and mitochondrial fusion, both of which are favorable for memory T cell development [76]. However, augmenting glycolytic metabolism by overexpressing the glycolytic enzyme phosphoglycerate mutase-1 drives T cells toward a terminally differentiated state and limits the formation of long-lived memory T cells, suggesting that a proper balance between glycolysis and mitochondrial metabolism is essential for supporting memory fate commitment [77].

Under conditions of chronic antigen stimulation, T cells gradually acquire a terminally exhausted phenotype, marked by reduced cytokine production and sustained expression of inhibitory receptors such as programmed cell death protein 1 (PD-1), LAG-3 and TIM-3. This process gives rise to two distinct subsets of exhausted T cells: progenitor exhausted T cells (Tpex) and terminally exhausted T cells (Tex) [78,79]. CD8^+^ Tpex and Tex exhibit distinct metabolic profiles, particularly in glucose metabolism, which significantly influence their differentiation and functional states. Tpex maintain a balanced metabolic state, utilizing both OXPHOS and glycolysis. This metabolic flexibility supports their self-renewal capacity and responsiveness to stimuli [80]. By contrast, Tex exhibit increased reliance on glycolysis to compensate for severely impaired OXPHOS, which is accompanied by mitochondrial fragmentation and reactive oxygen species (ROS) accumulation (Fig 2) [81]. Despite enhanced glycolysis, glucose uptake and metabolic function decline, resulting in insufficient energy production. This dual metabolic insufficiency leads to an “energy collapse” state, causing Tex to lose their proliferative capacity and effector functions, ultimately contributing to terminal exhaustion. Additionally, deletion of PKM2 diverts metabolic flux into the PPP, promoting Tpex accumulation and enhancing responses to PD-1 blockade in vivo [82]. These findings underscore the critical role of metabolic balance in the differentiation of exhausted T cells.

### Amino acid metabolism

In addition to carbohydrate metabolism, amino acid metabolism has vital and multifaceted roles in T cell survival, activation and function (Fig 2) [83]. Upon activation, T cells increase the expression of amino acid transporters and metabolic enzymes to meet elevated biosynthetic and energetic demands, a process driven by TCR signaling and regulated by key energy-regulating factors such as c-Myc, HIF-1α and mTOR [72,84,85].

Glutamine is a prime example of how amino acid metabolism supports T cell function [86]. Upon activation, T cells significantly increase glutamine uptake to fuel proliferation, effector differentiation and cytokine production [87,88]. Depletion of glutamine impairs these processes, leading to reduced T cell function [89]. Interestingly, limiting glutamine metabolism can also prevent T cell exhaustion and enhance memory T cell formation, promoting long-lasting anti-tumor responses [90]. These findings highlight the context-dependent role of glutamine metabolism in shaping T cell fate. Similarly, ʟ-arginine also supports the generation of central memory-like T cells by shifting metabolism from glycolysis to OXPHOS, thereby enhancing survival and anti-tumor efficacy [91]. Asparagine, although considered non-essential, is critical during T cell activation. Its deprivation disrupts protein synthesis and LCK phosphorylation, thereby impairing T cell activation [92]. Paradoxically, restricting asparagine availability can enhance CD8^+^ T cell metabolic fitness through activation the nuclear factor erythroid 2-related factor 2-dependent stress response or ROS-mediated metabolic and signaling adaptations, thereby promoting sustained proliferation and effector function [93]. These opposing effects illustrate how T cells’ nutritional demands evolve from activation to memory stages.

Additionally, amino acids such as methionine and cysteine modulate T cell responses in a stage-specific manner [11,94]. Collectively, these findings underscore the pivotal role of amino acid metabolism in shaping T cell immunity, offering promising therapeutic avenues to enhance immune responses through targeted metabolic interventions.

### Lipid metabolism

Effector T cells rely on de novo fatty acid synthesis for rapid proliferation, while memory T cells primarily utilize FAO and OXPHOS to support long-term survival and maintain a quiescent state (Fig 2) [95–97]. FAO induction in memory T cells is linked to AMPK activation, a key regulator of catabolic metabolism. Loss of the transcription factor TRAF6 disrupts AMPK signaling, impairing central memory CD8^+^ T cell differentiation. However, pharmacological activation of AMPK can restore memory T cell formation [98]. Carnitine palmitoyltransferase 1α (CPT1α) is essential for FAO and the mitochondrial import of long-chain fatty acids [99]. Elevated CPT1α expression supports the generation of memory CD8^+^ T cells, with cytokines such as IL-15 enhancing mitochondrial biogenesis and upregulating CPT1α [100]. However, the precise role of FAO in memory T cell formation remains debated, as FAO inhibition impairs memory development, yet CPT1α deletion does not replicate this defect.

Dysregulated lipid metabolism in the TME impairs T cell function and promotes immune evasion. Excessive accumulation of lipids such as cholesterol negatively impacts CD8^+^ tumor-infiltrating lymphocytes (TILs). Fatty acid uptake through CD36 induces lipid peroxidation, activating the p38–MAPK pathway or ferroptosis, leading to T cell dysfunction. However, interventions such as CD36 ablation, p38 inhibition or glutathione peroxidase 4 overexpression can restore T cell function [101,102]. In pancreatic cancer, abnormal accumulation of long-chain fatty acids disrupts mitochondrial function by downregulating very-long-chain acyl-CoA dehydrogenase, impairing T cell activity [103]. In metastatic ovarian cancer, TME-induced endoplasmic reticulum stress reduces transgelin-2 expression in CD8^+^ TILs, impairing lipid uptake and T cell function. Restoring transgelin-2 expression enhances lipid uptake, mitochondrial respiration and cytotoxic capacity [104]. Dysregulated lipid metabolism within the TME presents a promising target for immune-based therapies. Therapeutic strategies aimed at enhancing FAO in memory T cells or limiting lipid accumulation in TILs may help optimize T cell responses and improve the efficacy of cancer immunotherapy.

### Metabolic characteristics of other subsets T cells

While our previous discussion has focused on the metabolic characteristics of CD8^+^ T cells in anti-tumor immunity, it is equally important to consider the metabolic programming of other T cell subsets that regulate anti-tumor immune responses within the TME. In particular, CD4^+^ T cell subsets, such as Tregs and T helper 17 (TH17) cells, play distinct roles in shaping anti-tumor responses [25,105–107].

Tregs are indispensable for maintaining immune tolerance and are often co-opted by tumors to suppress antitumor immunity [17]. These cells predominantly rely on OXPHOS and FAO to sustain their survival and immunosuppressive functions under the nutrient-poor and often hypoxic conditions of the TME (Fig 2) [108]. The metabolic programming of Tregs is tightly regulated by TCR signaling and mTOR activity. Notably, deletion of the catalytic subunit of glutamate cysteine ligase in Tregs leads to increased serine metabolism and mTOR activation but downregulates transcription factor FOXP3 expression, impairing suppressive function [108]. Furthermore, the glycolytic enzyme enolase-1 directly binds the FOXP3 promoter, and its deletion increases FOXP3 expression [109], highlighting a tight link between metabolism and Treg stability.

By contrast, TH17 cells adopt a markedly different metabolic profile characterized by a strong reliance on aerobic glycolysis and glutaminolysis to fuel their rapid proliferation and pro-inflammatory effector functions. These metabolic shifts not only meet the bioenergetic and biosynthetic demands of TH17 cells but also critically shape their differentiation and function [110–116]. Pharmacological targeting of TH17 cell metabolism holds therapeutic promise. For example, the antimalarial drug artesunate inhibits IRF4-dependent glycolysis in both murine and human TH17 cells, suppressing their proliferation and differentiation [115]. In addition, overexpression of glycolytic regulators such as PKM2 or the glucose transporter GLUT-3 enhances glycolytic flux, fine-tunes transcription factor STAT3 signaling, and drives glycolysis-dependent epigenetic remodeling, thereby reinforcing TH17 cell differentiation [116,117]. Intriguingly, recent studies have uncovered a sex-specific layer of metabolic regulation. In models of allergic airway inflammation, androgen signaling attenuates glutaminolysis and thereby promotes TH17 cell differentiation [112], uncovering a previously underappreciated link between hormonal cues and TH17 cell differentiation and suggesting novel, sex-specific immunomodulatory strategies.

Given the shared ontogeny and plasticity of Tregs and TH17 cells, a central question is how environmental metabolic cues, such as nutrient availability, hypoxia and immunosuppressive metabolites, tip the balance between these two subsets in the TME. Elucidating this metabolic reprogramming will not only shed light on the mechanisms governing T cell fate decisions but also identify strategies to reprogram CD4^+^ T cell responses in favor of effective anti-tumor immunity.

## How metabolic reprogramming of cancer cells and T cells affects tumor progression

Metabolic reprogramming in cancer cells and T cells drives tumor progression by reshaping the TME, altering nutrient availability and influencing immune cell function. In this section, we focus on the metabolic crosstalk between cancer cells and T cells, highlighting how this bi-directional interaction contributes to tumor development and immune regulation.

### The impact of tumor cell metabolic reprogramming on T cells

#### Nutrition competition in TME.

**Fig 3:**
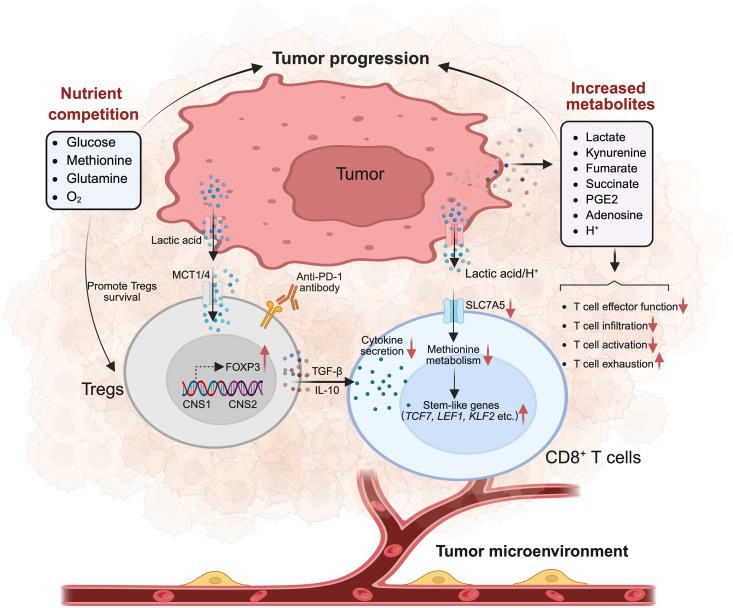
Metabolic interplay of cancer cells and T cells in the TME. The tumor microenvironment (TME) is enriched with immunosuppressive cell populations such as regulatory T cells (Tregs), which promote tumor progression and suppress anti-tumor immunity through metabolic reprogramming and the secretion of inhibitory factors, such as TGF-β and IL-10. Tumor cells further contribute to immune dysfunction by outcompeting effector T cells for critical nutrients, including oxygen, glucose and amino acids, thereby impairing T cell infiltration, effector function and cytotoxicity. Moreover, the accumulation of tumor-derived metabolites such as lactate, which suppresses T cell proliferation and function, yet may support T cell stemness, profoundly reshapes the immune landscape. Additional metabolites, including kynurenine, fumarate and succinate, further contribute to CD8^+^ T cell dysfunction and exhaustion. Collectively, these factors establish a metabolically hostile and immunosuppressive milieu that impairs effective antitumor immune responses. MCT, monocarboxylate transporter; PD-1, programmed cell death protein 1; PGE2, prostaglandin E2. Figure created with BioRender, https://www.biorender.com.

In the TME, both tumor cells and immune cells face substantial energy demands. Tumor cells meet their growth requirements while simultaneously suppressing the effector functions of immune cells through intense nutrient competition. Tumors directly disrupt key metabolic pathways in effector immune cells by outcompeting them for critical nutrients. For instance, glucose and oxygen consumption by tumor cells can limit T cell function, leading to reduced mTOR activity, diminished glycolytic capacity and impaired IFN-γ production [118,119] (Fig 3).

Competition for amino acids by tumor cells also exacerbates this effect. In lung squamous cell carcinoma, tumor cells deplete methionine, an essential precursor required for T cell activation, ultimately reducing T cell cytotoxicity and increasing the proportion of exhausted T cells [120]. In breast cancer, elevated glutamine uptake by tumor cells inhibits T cell-mediated cytotoxicity [121]. In cervical cancer, tumor cells deplete serine from the TME via serine incorporator 2 expression, leading to T cell exhaustion, reduced effector function, and cell death [122]. These nutrient-driven competitions exclude T cells from tumor tissues, limiting their infiltration, function and tumor-killing capabilities.

#### Tumor-secreted metabolites foster a suppressive TME.

The TME is rich in metabolic byproducts secreted by tumor cells, which have a profound impact on T cell function (Fig 3). Lactic acid, a major byproduct of aerobic glycolysis in tumor cells, impairs T cell proliferation and cytokine production. Lactic acid consists of lactate and hydrogen ions, which lower the pH of the surrounding environment. Recent studies have demonstrated that short-term exposure to low pH inhibits T cell infiltration by downregulating integrin β1 expression via suppression of the METTL3–N6-methyladenosine (m6A) axis [7,15]. However, prolonged exposure to low pH can induce a transition of T cells toward a stem-like phenotype by altering methionine metabolism, ultimately enhancing T cell immune responses [123]. This finding may explain the presence of TCF-1^+^ stem-like T cells, critical immune checkpoint responders, both in tumors and draining lymph nodes. Lactate also plays a crucial role in T cell differentiation. Studies have shown that adding high-concentration sodium lactate to in vitro cultures promotes stem-like differentiation of T cells by inducing H3K27 acetylation at the *TCF7* locus [124]. These findings suggest that lactate and hydrogen ions influence T cell differentiation and function through distinct mechanisms. The effects of lactic acid on T cells are highly context-dependent, with its availability and environmental conditions influencing T cell fate. For instance, in the presence of repeated tumor antigen stimulation, lactic acid promotes T cell exhaustion. Conversely, prolonged exposure to lactate without antigen stimulation drives T cell stem-like differentiation.

In addition to lactic acid, other tumor-derived metabolites profoundly influence T cell immunity. Kynurenine, a key product of tumor-associated tryptophan metabolism, is absorbed by T cells and promotes T cell exhaustion [125,126]. Another immunosuppressive factor, fibroblast growth factor 21, sustains hyperactivation of the AKT–mTORC1–SREBP1 signaling axis in activated CD8^+^ T cells, leading to enhanced cholesterol biosynthesis and T cell exhaustion [127]. Prostaglandin E2, an unsaturated fatty acid secreted by tumor cells, suppresses T cell responses both indirectly by modulating inflammatory monocytes and directly by reducing T cell sensitivity to IL-2, thereby impairing their anti-tumor activity [128–130]. Notably, tumor-derived fumarate accumulates in the TME and covalently modifies ZAP70 through succination, further disrupting TCR signaling and impairing T cell activation [131]. These findings illustrate the intricate ways in which tumor-derived metabolites subvert T cell immunity. Targeting these metabolic pathways holds great promise for counteracting immune suppression and enhancing antitumor responses.

### The impact of T cell metabolic reprogramming on tumor progression

T cell metabolic reprogramming not only governs T cell differentiation and function, but also profoundly influences tumor progression by reshaping the metabolic and immunologic landscape of the TME [132–135].

Tregs predominantly depend on OXPHOS and FAO, enabling them to survive and maintain immunosuppressive function in the nutrient-poor, hypoxic TME, thereby facilitating tumor growth by limiting cytotoxic T lymphocyte activity and promoting immune evasion [108,136]. Moreover, Tregs intensify metabolic competition by consuming essential nutrients such as glucose, further impairing effector T cell function. Notably, intra-tumoral Tregs can up-regulate glycolytic pathways that allow them to utilize lactic acid as an alternative energy source, thereby supporting their proliferation and suppressive function [26]. In addition, Tregs secrete immunosuppressive molecules such as adenosine and IL-10, directly contributing to tumor progression [134,137–139].

TH17 cells play a dual role in tumor immunity. On one hand, TH17-derived cytokines, such as IL-17A and GM-CSF, can enhance anti-tumor immunity by activating dendritic cells and recruiting CD8^+^ T cells [140–142]. On the other hand, under certain metabolic or pro-inflammatory conditions, TH17 cells can produce the cytokine TWEAK, which interacts with its receptor Fn14 to drive EMT, migration and invasion [143]. Furthermore, chronic exposure to IL-17A may promote angiogenesis, EMT and proliferation in inflammation-driven tumors [144–147]. Intriguingly, the functional plasticity of TH17 cells is closely linked to their metabolic state. Inhibiting IRF4-driven glycolysis or modulating glutaminolysis can reprogram TH17 cell cytokine profiles, reducing their pro-tumorigenic effects while enhancing immune-stimulatory phenotypes [115]. This metabolic plasticity offers opportunities to selectively preserve the beneficial properties of TH17 cells while mitigating their tumor-promoting potential. In summary, the metabolic programming of Tregs and TH17 cells reshapes tumor progression. Targeting their metabolic dependencies, either by disrupting Treg fitness or reprogramming TH17 cell metabolism, offers a promising strategy to remodel the tumor ecosystem to enhance anti-tumor immunity.

## Applications of metabolic reprogramming in cancer therapy

Targeting the altered metabolic states of both tumor cells and T cells has emerged as a promising strategy in cancer therapy. By modulating key metabolic pathways, such as glycolysis, glutamine metabolism and FAO, researchers aim to overcome tumor resistance and enhance the effectiveness of immunotherapies. Exploiting the metabolic dependencies of cancer cells opens new avenues for treatment, while reprogramming T cell metabolism can improve their functionality and longevity. This section highlights current applications and emerging directions in metabolic interventions for cancer therapy.

### Leveraging metabolic dependencies of cancer cells to enhance treatment efficacy

Targeting metabolic pathways such as glycolysis, FAO and glutamine metabolism has emerged as a promising cancer treatment strategy. Several small-molecule inhibitors, including GLUT inhibitors (e.g., fasentin, WZB117) and nicotinamide phosphoribosyltransferase inhibitors (e.g., GMX1778, STF-31), impair tumor cell proliferation by disrupting glucose uptake and energy production [148,149]. Other compounds targeting key glycolytic enzymes (such as 2-deoxyglucose and 3-bromopyruvate, which are HK2 inhibitors, and PKM2-IN-1, a PKM2 inhibitor) have shown anti-tumor effects in preclinical models [150]. Notably, some regulators that alter these glycolytic enzymes have the potential to be developed into pharmaceuticals. For instance, protein phosphatase 4 regulator subunit 1 upregulates tumor glycolysis through strengthening the interaction between ERK1/2 and PKM2, promoting the growth and metastasis of gallbladder cancer [151]. The epigenetic modification enzyme METTL3 up-regulate the expression of HK2 through m6A modification in mRNA, enhances glycolysis in pancreatic ductal adenocarcinoma cells and promotes invasion [152]. However, due to the widespread expression of these metabolic enzymes in normal proliferating cells, therapeutic application is limited by toxicity. Moreover, treatments such as programmed death ligand 1 (PD-L1) blockade can indirectly suppress tumor glycolysis via mTOR inhibition, highlighting the complex interplay between metabolism and immune regulation [118].

Beyond glycolysis, tumors can utilize OXPHOS for ATP generation. Mitochondrial complex I, the entry point of the electron transport chain, not only drives bioenergetic flux but also regulates ROS, supports immune evasion and promotes metastasis, making it a promising metabolic target for cancer therapy [153–155]. Metformin, a widely prescribed anti-diabetic drug, exerts a mild inhibitory effect on mitochondrial complex I. Epidemiological studies have linked metformin use to improved cancer outcomes, yet clinical benefits appear limited to subsets of patients (e.g., those with HER2^+^ breast cancer), likely reflecting inter-tumor metabolic heterogeneity. Moreover, IACS-010759, a more potent and selective mitochondrial complex I inhibitor, has shown promising anti-tumor activity but was associated with significant adverse events in early trials [156–159]. Therefore, additional clinical studies are required to fully assess both the efficacy and safety of targeting mitochondrial complex I.

Recent studies have revealed that targeting glucose metabolism alone may be insufficient for durable cancer control, as tumor cells can adapt by reprogramming their metabolism to rely on fatty acids and amino acids for ATP production. Consequently, interventions targeting lipid and amino acid metabolism have gained attention as complementary therapeutic strategies. For instance, etomoxir, an inhibitor of mitochondrial FAO, suppresses tumor growth and reduces CD47-mediated immune evasion in glioblastoma multiforme [160]. Similarly, the glutamine transporter inhibitor V-9302 selectively blocks glutamine uptake in TNBC cells, thereby impairing cancer cell proliferation [121]. In addition, enzymes involved in amino acid catabolism, such as indoleamine 2,3-dioxygenase (IDO) in the kynurenine pathway, play critical roles in tumor-associated immunosuppression by depleting tryptophan and accumulating immunosuppressive metabolites. While preclinical studies suggested that IDO inhibition could enhance the efficacy of immune checkpoint therapies such as anti-PD-L1, clinical translation has proven challenging. Notably, the IDO1 inhibitor epacadostat failed to demonstrate clinical benefit in a phase III trial when combined with immune checkpoint blockade in patients with unresectable or metastatic melanoma [139,161–163]. These findings underscore the metabolic adaptability of tumors and highlight the necessity for thorough preclinical characterization and clinical validation when advancing metabolic targets toward therapeutic applications.

Although advances in cancer metabolism research are encouraging, many questions remain. Ideal metabolic therapies should selectively target tumors without harming normal or immune cells. To address undruggable targets, technologies such as proteolysis targeting chimeras (known as PROTACs) offer promise by degrading proteins via E3 ubiquitin ligase recruitment. For example, ARV-110, which targets the androgen receptor, has shown efficacy in phase II trials [164]. However, the limited availability of suitable E3 ligases presents a technical barrier. Thus, continued exploration of tumor metabolic networks and innovative therapeutic platforms is essential to translate these insights into effective and precise cancer treatments.

### Leveraging T cell metabolic programming for enhanced cancer immunotherapy

Adoptive T cell therapy, encompassing strategies such as chimeric antigen receptor T (CAR-T) cell, T cell receptor-engineered T cell and TIL therapies, has emerged as a promising modality for cancer treatment. However, the metabolic challenges imposed by the TME often undermine the persistence and effector functions of these adoptively transferred cells. Consequently, metabolic reprogramming is being actively explored as a means to enhance the therapeutic efficacy of adoptive cell therapy.

#### CAR-T cell metabolism and costimulatory domains.

CAR-T cell therapy involves genetically modifying T cells to express chimeric antigen receptors that enable precise tumor recognition and elimination. Critically, the intracellular signaling domains of CAR constructs directly influence the metabolic program of these engineered cells. For example, CD28 costimulatory signaling enhances glycolysis, which supports rapid effector functions, whereas 4-1BB signaling promotes mitochondrial biogenesis and helps maintain oxidative metabolism, thereby contributing to prolonged cell persistence [164,165]. The most effective CAR constructs often incorporate both CD28 and 4-1BB domains, creating a metabolic balance that fosters both immediate effector responses and long-term survival. Emerging strategies have also examined the inclusion of additional costimulatory molecules. One recent study demonstrated that incorporating the B cell costimulatory molecules CD79A and CD40 into CAR-T cells not only enhances glycolytic activity but also markedly improves anti-tumor responses [166]. Nevertheless, the selection of optimal costimulatory combinations is complex; rather than simply adding signals, the goal is to maximally remodel T cell metabolism in a synergistic manner, a challenge that requires further experimental validation and design refinement.

#### Genome editing and engineering to modulate T cell metabolism.

Genome editing tools are being leveraged to directly target metabolic pathways in T cells. For instance, complete knockout of meteorin-like, a cytokine secreted in the TME that disrupts CD8^+^ T cell metabolism by promoting mitochondrial dysfunction, enhances metabolic fitness and improves tumor control [167]. Similarly, adenosine, a potent immunosuppressive metabolite that acts via the A2A receptor (A2AR), limits T cell activity. A2AR deletion or engineered overexpression of adenosine deaminase, which converts adenosine into inosine, effectively overcomes adenosine-induced immunosuppression in CAR-T cells, thereby enhancing cytokine production and anti-tumor activity [168–170]. Additionally, given the role of O-GlcNAc transferase in sustaining T cell “stemness” through mannose-mediated signaling [171], engineering T cells to express higher level of O-GlcNAc transferase represents another promising strategy.

#### *Ex vivo* metabolic preconditioning of therapeutic T cells.

One of the major hurdles in adoptive cell therapy is preventing ex vivo expanded T cells from terminally differentiating into short-lived effector cells. Instead, maintaining a “stem-like” state is crucial for persistent anti-tumor function after infusion. The ex vivo expansion phase provides an ideal window for metabolic intervention. Supplementing culture media with specific metabolites or cytokines can favorably modulate T cell metabolic profiles. For example, IL-15 enhances OXPHOS and FAO, thereby supporting long-term T cell survival [172], while IL-7 can promote a balanced increase in both glycolysis and OXPHOS [173].

Recent studies have also explored the addition of lactic acid or lactate salts to culture conditions. Although the effects of lactate on T cells remain a subject of debate, its incorporation has been observed to enhance the “stemness” of CAR-T cells, ultimately boosting their anti-tumor efficacy [123,124]. A recent study has shown that D-mannose, a cost-effective monosaccharide, more effectively promotes a stem-like phenotype than conventional IL-15 treatment, while also supporting cell expansion for up to several months. D-mannose significantly increased TCF1 expression, promoted T cell differentiation toward a less differentiated state and enhanced anti-tumor activity, leading to prolonged survival in preclinical models [171]. The ability of D-mannose to support long-term culture and maintenance of T cell states holds great promise for both the expansion of TILs and the generation of standardized, universal CAR-T cell products.

#### Combined metabolic and checkpoint blockade strategies.

In addition to restoring intrinsic T cell activity, immune checkpoint blockade therapies also exert metabolic regulatory effects. For instance, PD-1 blockade can reprogram T cell metabolism by downregulating glycolysis while enhancing FAO, thereby improving cell persistence [174]. Combining immune checkpoint blockade with metabolic interventions may, therefore, produce synergistic benefits. For example, metformin reprograms tryptophan metabolism, stimulating CD8^+^ T cell cytotoxicity; its combination with anti-PD-1 therapy enhances CD8^+^ T cell proliferation and IFN-γ production [175,176]. Similarly, the antifolate agent pemetrexed can induce immunogenic cell death, increase cytotoxic T cell infiltration, and augment the anti-tumor effects of PD-1 blockade [177,178]. A recent study has shown that D-mannose enhances the effectiveness of immunotherapy and radiotherapy in TNBC by promoting the degradation of PD-L1 [179]. This dual function, which both inhibits tumor growth and boosts T cell activity, positions D-mannose as a highly promising therapeutic agent.

While these metabolic interventions offer numerous promising avenues to bolster adoptive T cell therapy, future research must focus on optimizing the combination and timing of these strategies. A key objective is to maximize T cell persistence and function while mitigating the immunosuppressive challenges of the TME. In parallel, reducing the cost and complexity of metabolic interventions will be essential for their translation into clinical practice. As our understanding of metabolic networks deepens, particularly through high-resolution metabolic profiling and genetic screening, new targets and combinatorial approaches will likely emerge, further advancing the field of cancer immunotherapy.

## Conclusion

The complex metabolic interactions between cancer cells and T cells within the TME critically influence immune function and tumor progression through mechanisms such as nutrient competition and metabolic reprogramming [7,30,93,129]. The outcomes of these processes are governed not only by nutrient availability but also by intrinsic biochemical and biophysical properties of each cell type, such as transporter affinity [180,181], metabolic flexibility and adaptability to immunosuppressive niches [17,182–184], ultimately resulting in an imbalanced metabolic partitioning. Targeting key metabolic pathways, including aerobic glycolysis, glutamine metabolism and fatty acid metabolism, can disrupt tumor immune evasion [185,186], induce cancer cell apoptosis [37,187] and enhance sensitivity to immunotherapies [188–190], highlighting the promising potential of metabolic interventions. Combining metabolic inhibition with immunotherapy, such as modulating T cell metabolism and reprogramming immunosuppressive cells, offers an effective strategy to restore tumor-fighting immune responses, suppress tumor-supportive cells and foster a more favorable anti-tumor environment. However, the high metabolic plasticity of tumor cells enables them to outcompete other cells for nutrients and actively reshape the TME to their advantage. This not only suppresses the activity of cytotoxic CD8^+^ T cells but also promotes the accumulation of exhausted CD8^+^ T cells and immunosuppressive Tregs. In addition, tumor cells actively secrete immunosuppressive cytokines such as TGF-β, which directly inhibit T cell effector functions. Furthermore, lactate is not merely a passive consequence of metabolic overflow; its accumulation contributes to an immunosuppressive milieu and may actively impair T cell function and viability [191–194].

Despite significant advances in our understanding of how tumor cells and T cells rewire their metabolism, translating these insights into effective therapies remains challenging. The TME comprises heterogeneous tumor subclones and diverse T cell subsets, each with unique metabolic dependencies, complicating the identification of optimal therapeutic targets. Moreover, many metabolic inhibitors designed to starve cancer cells simultaneously impair effector T cells that rely on similar pathways, thereby compromising immune function [195,196]. Metabolic profiles also vary widely across tumor types and disease stages, limiting the broad applicability of single-agent interventions.

To overcome these obstacles, several key strategies are required. For example, high‐resolution techniques, such as single‐cell and spatial metabolomics, should be employed to generate comprehensive metabolic maps of all cellular constituents within tumors, thereby identifying unique metabolic vulnerabilities. In addition, therapeutic delivery systems, optimized in terms of dosing, timing and vehicle design, are needed to target metabolic inhibitors specifically to tumor cells or immunosuppressive subsets (e.g., Tregs), while sparing effector T cells. Furthermore, rational combination regimens that integrate metabolic modulators with immune checkpoint inhibitors or adoptive cell therapies should be developed to synergistically enhance anti-tumor responses. By implementing these approaches, it may be possible to achieve precision metabolic interventions that both suppress tumor growth and preserve or even boost host immune function, ultimately improving clinical outcomes.

## Abbreviations

ACSL4: acyl-coenzyme A synthetase long-chain family member 4
AMPK: AMP-activated protein kinase
ATP: adenosine triphosphate
CAR-T: chimeric antigen receptor T
CPT1α: carnitine palmitoyltransferase 1 α
CRC: colorectal cancer
EMT: epithelial–mesenchymal transition
FAO: fatty acid oxidation
FBP2: fructose-1,6-bisphosphatase 2
GDP: guanosine diphosphate
GLUT-1: glucose transporter 1
HIF-1α: hypoxia-inducible factor 1α
HK2: hexokinase 2
IDO: indoleamine 2,3-dioxygenase
LDHA: lactate dehydrogenase A
m6A: N6-methyladenosine
OXPHOS: oxidative phosphorylation
PD-1: programmed cell death 1
PKM2: pyruvate kinase M2
PPP: pentose phosphate pathway
PROTACs: proteolysis targeting chimeras
ROS: reactive oxygen species
TCA: tricarboxylic acid
TCR: T cell receptor
Tex: terminally exhausted T cells
TH17: T helper 17
TILs: tumor-infiltrating lymphocytes
TME: tumor microenvironment
TNBC: triple-negative breast cancer
Tpex: progenitor exhausted T cells
Tregs: regulatory T cells
